# Philadelphia Letter

**Published:** 1881-01

**Authors:** 


### Domestic Correspondence.

### ARTICLE XI.

### PHILADELPHIA LETTER.

MESSRS. EDITORS:—A glance at the social aspects of professional life in this city may perhaps not lack interest for your readers, and although your correspondent cannot at this time enter into detail, nor indeed do more than touch upon one or two points of this subject, yet to the intelligent man, and especially to the physician, the life of his professional brethren in a distant city cannot be altogether uninteresting, and may in some aspects be highly instructive by example or warning.

One of the characteristics of professional life here in Philadelphia is our comparative freedom from those bitter feuds and heart-burning jealousies which prevail in other large cities, where perhaps there may be more inequality of success than with us, and where the race for wealth alone is more reckless and furious than with our comparatively cool and calm citizens.

But if we are comparatively free from some of the faults of our neighbors, we suffer from others to which perhaps they are less subject. Possibly we are too prone to underrate our home talent, and I have heard it said that in Philadelphia a reputation is the echo of foreign rumor, and that it is not until a man is the recipient of honors from distant parts that his fellow-townsmen begin to wake up to the fact that So-and-so has more in him than any one suspected. Our Quaker progenitors are answerable for some of this feeling; they used to put down with a firm hand any attempt at show, and especially if this was not backed by substance. When I was a medical student, a rather seedy-looking old gentleman used to haunt the hospital, of whom the story was told that his professional life had been blighted by the severity of the "Friends." It was said that on entering into practice, young Benjamin, who was a "Friend," assumed a modest but stylish turn-out, with which he could be seen driving rapidly up and down the streets of the Quaker city, then (for it was sixty years ago) much quieter and smaller than now, and attracted the attention of the good people going to "meeting" by the evidently thriving practice the young man enjoyed. In a few weeks, however, the young man was waited upon by two solemn brethren in portentously broad brimmed hats, who said, "Benjamin, the meeting understands that thee hast been seen of late driving about the streets of this town in a new and expensive vehicle called a gig. Now thee must know that the friends are aware thee hast no need of such a conveyance, and that thee canst ill afford the expense thereof. We therefore admonish thee that thou at once give up this idle display, or thee wilt render thyself liable to discipline." The gig was given up, but the poor young doctor never recovered from the blow, and though gaining some reputation, he never gained sufficient practice to warrant the purchase of a vehicle. There in his office, where the old broad-brims admonished him, he has lived all these years, and as I sometimes pass the little old-fashioned house away down town, and see the battered sign with the letters long illegible, and the gray-haired old gentleman sitting inside, I think of this story current among the hospital students, and think that if it wasn't true it was typical.

Perhaps, however, though this democratic levelling and universal combination against undue pretension has its evil and narrowing side, yet it also has its advantages, and perhaps not least among these is that it is conducive to social amenities among the members of the profession at large. We have clubs of all kinds, large and small. Book clubs for the dissemination of medical literature which, especially in the outlying parts of the city, take on a social aspect and perhaps have a club-room and a library made up of the volumes which have circulated among the members. Some of these clubs and associations have festal gatherings from time to time, when the enjoyment is of a material sort; others meet to read papers or invite some big gun or specialist to read a paper before them. Then there are other circles of a purely festive sort, meeting regularly week by week at the houses of the various members. An attempt at social enjoyment on a larger scale has been initiated this season, in the form of a series of receptions given by the president of the County Medical Society. This is our largest and most active society, and is truly representative. Here one may see the semi-rural practitioner, with mud-splashed boots, sitting cheek by jowl with the elegant and polished consultant, and the newly-fledged graduate drinking in the utterances of the professor who is holding forth on his specialty or his hobby. There is no better place to feel the pulse of the profession of Philadelphia, and no surer and quicker road to practice than is found there by those who show themselves the possessors of that sort of knowledge which the average practitioner wants to call upon in his hour of need.

Dr. Albert H. Smith, the well-known gynæcologist, is the present president of the "County Medical," and his receptions have been a marked success, and must redound to the advantage of the society. Not only all the members of the society have been at one time or another present at these agreeable reunions, but many outsiders who have been invited have found for the first time the advantage of meeting representative men, and have been induced to such membership, so that the aim of its promoters to limit the membership of the society only by the number of practitioners in the county, seems more likely of realization than one might suppose.

The medical profession here has been honored in the choice of one of its members to preside over our chief seat of learning, the University of Pennsylvania, Dr. William Pepper, Professor of Clinical Medicine in the Medical Department, having been recently elected by the trustees "Provost" of the University.

Such an honor does not often fall on so young a head (the new Provost is barely thirty-seven), and it is indeed rare to find among our busy and "practical" medical men the high general culture requisite to sustain this dignified position. But Dr. Pepper is no ordinary man. From early youth he has been marked as one likely to achieve more distinction than falls to the lot of men of average ability. At college I am told that he was easily first in everything, graduating at the head of his class and carrying off all the prizes in every branch. Not very long after his graduation in medicine—at the early age of twenty-four, in fact—he obtained the position of attending physician to the medical department of the City Hospital, having at least 200 beds under his control. Here Dr. Pepper laid the foundation of the extensive and accurate clinical knowledge for which he is so distinguished. Every morning, punctually at half past seven, he was accustomed to make his appearance in the ward, and accompanied by the resident physician, who was expected to take full notes from his dictation, he went from bed to bed, working thus until nine, when he left the wards and spent an hour in the post-mortem room, there also dictating copious notes. His clinical lectures, even at that time, were models of terse and flowing English. A rapid speaker, never hesitating for a word, his utterance singularly clear and musical, he rivetted the attention of his audience, and aside from the value of his teaching delighted those who had an ear for delicate shades of meaning by choosing instinctively the exact word or expression which would best convey the idea. I speak in the past tense because my knowledge of Dr. Pepper as a hospital physician and lecturer is confined to that period of his career, and I have rarely heard him lecture of late years, although I have no doubt he still maintains the high reputation made at that time.

His first literary work was in connection with Dr. J. Forsyth Meigs, of this city, in getting out a new edition of this gentleman's work on diseases of children. It is an open secret that the major part of this edition, which was in reality almost a new book, was written by Dr. Pepper. The reputation it has made is very great, and "Meigs and Pepper" has become one of the standard works on the subject of which it treats. But Dr. Pepper's genius shows more in the executive ability which he displayed in reanimating the moss-grown and decrepit medical department of the University. Ten or twelve years ago this had fallen into a mouldy decline. Content to dwell in the glories of the past, the Professors appeared careless of the present reputation of the institution under their charge, which was rapidly falling hopelessly behind the age. At that time, so meagre was the clinical instruction afforded that students have been known to graduate without ever having felt a pulse or touched a sick person.

Out of this slough, Dr. Pepper, aided by a few other young men, dragged the palsied and decrepit *Alma Mater*, set her on her legs, and not merely galvanized her into life, but by a judicious transfusion of new blood, rejuvenated her and placed her in the first rank among American medical institutions. The man who could do that was evidently the man to raise the whole University, and the election of Dr. Pepper to the Provostship has been hailed by all the friends of the University, one of whom, a wealthy merchant, has just given $50,000 for the purpose of building a new wing for the University Hospital. One of our morning papers compared Provost Pepper's proposed work to the labors of Hercules, but we have in him a Hercules of sufficient energy to do the work. His device, which I happened to see in a book-plate in one of his books, is a thorn-bush with the motto of Sainte Aldegonde, one of the noblest spirits who ever lived, "*Repos Ailleurs.*" And this active spirit which, not content with the duties of hospital physician and Professor of Clinical Medicine, with a large practice, desires for itself more occupation, will doubtless succeed in this task, also, as in all others hitherto undertaken.

Among smaller matters of local interest I may mention the appearance of a joint work on typhoid fever by Prof. Da Costa and Dr. Ellwood Wilson. The former, who is getting out a new edition of his famous work on diagnosis, is too well known to need further mention. Wilson has only a local reputation ; he is, perhaps, the most fashionable doctor in town ; for a Philadelphia woman to lie-in without Wilson in attendance, is to forfeit the consideration of society. Prof. H. C. Wood has just gotten out a work on diet in disease, in collaboration with Dr. J. Milner Fothergill, of London. The second edition of Prof. Duhring's " Treatise on Diseases of the Skin ", will be out in a few weeks ; it is like a new work, the author having revised every page with the painstaking care characteristic of himself.

Your readers who have followed the history of the notorious " Dr." Buchanan, will be interested in hearing that he has been fined $500 and sentenced to imprisonment for ten months. The " mill " has been permanently closed, its charter having been revoked.

